# Titin Cardiomyopathy Associated With Refractory Ventricular Tachycardia: A Case Report

**DOI:** 10.7759/cureus.64476

**Published:** 2024-07-13

**Authors:** Aleksan Khachatryan, Justin Brilliant, Ashot Batikyan, Timm Dickfeld, Margarita Sargsyan, Vahagn Tamazyan, Joel Alejandro, Hakob Harutyunyan

**Affiliations:** 1 Department of Internal Medicine, University of Maryland Medical Center, Midtown Campus, Baltimore, USA; 2 Department of Cardiovascular Disease, University of Maryland Medical Center, Baltimore, USA; 3 Department of Internal Medicine, North Central Bronx Hospital, New York, USA; 4 Department of Cardiology, "Heratsi" Hospital Complex № 1, Yerevan, ARM; 5 Department of Internal Medicine, Maimonides Medical Center, New York, USA

**Keywords:** premature ventricular contractions, nonischemic cardiomyopathy, danicamtiv, left ventricular outflow tract obstruction (lvoto), right ventricular outflow tract, titin gene truncating variants, ventricular tachycardia, dilated cardiomyopathy (dcm), titin gene, titin

## Abstract

Cardiomyopathy is defined as structural and functional myocardial abnormality not attributed to ischemic, valvular, hypertensive, or congenital cardiac causes. The main phenotypes of cardiomyopathy include hypertrophic, dilated, non-dilated left ventricular, restrictive, arrhythmogenic right ventricular, Takotsubo, and left ventricular noncompaction cardiomyopathies. A significant proportion of dilated cardiomyopathy (DCM) cases represents patients with genetic mutations, most commonly titin gene truncating variants (TTNtv). It has been shown that TTNtv mutation contributes to the development of certain types of DCM such as alcohol, chemotherapy, and peripartum. We present a case of DCM where genetic workup revealed TTNtv without other contributing factors. The course was complicated by multiple ventricular tachycardias (VTs) refractory to medical management, despite treatment with amiodarone, sotalol, dofetilide, mexiletine, and propranolol. Interestingly, endocardial mapping failed to delineate the substrate of tachycardia. This report underscores the importance of genetic testing in DCM and highlights the potential association of titin cardiomyopathy with refractory VTs, possibly of epicardial origin.

## Introduction

Cardiomyopathy (CMP) is characterized by structural and functional abnormalities in the myocardium that are not caused by ischemic, valvular, hypertensive, or congenital cardiac conditions. There is no universally recognized classification of cardiomyopathies. According to the most recent update from the European Society of Cardiology, the major morpho-functional phenotypes include hypertrophic, dilated, non-dilated left ventricular, restrictive, arrhythmogenic right ventricular, Takotsubo, and left ventricular noncompaction cardiomyopathies [1].

Dilated cardiomyopathy (DCM) is defined by an impaired systolic function of the left or both ventricles, resulting in ventricular dilation secondary to genetic or non-genetic etiologies. Approximately 40% of DCM cases result from various genetic mutations [2]. Commonly detected genes include beta myosin heavy chain, alpha myosin heavy chain, cardiac troponin T, titin (TTN), alpha-tropomyosin, and cardiac troponin C. The inheritance pattern is typically autosomal dominant, although autosomal recessive, X-linked, and mitochondrial patterns have also been identified. Titin gene truncating variants (TTNtv), caused by missense or "stop" mutations, interrupt titin protein production and represent the most common genetic cause of DCM [3]. TTNtv mutations have been identified in approximately 10-15% of cases of alcohol-related, peripartum, or chemotherapy-associated DCM [4-6]. Previously considered solely attributable to external factors, these cardiomyopathies are now recognized to result from a combination of TTNtv mutations and environmental exposures in certain patients.

It has been shown that TTNtv-related DCM is associated with an increased risk of atrial and ventricular arrhythmias [7]. In our case, there were no predisposing factors or a family history of DCM. Interestingly, the source of refractory ventricular tachycardia (VT) remained unidentified on endocardial mapping highlighting the potential importance of performing both endocardial and epicardial mapping in cases of refractory VT.

## Case presentation

A 23-year-old male with a medical history of morbid obesity (BMI 46.68 kg/m²), nonischemic cardiomyopathy (NICMP), VT, status post right ventricular outflow tract (RVOT) ablation, deep venous thrombosis, and stroke presented with dyspnea, chest pain, and extreme fatigue.

The patient reported progressively worsening symptoms over the past two weeks, severely limiting his ability to perform even minimal activities of daily living due to dyspnea. His chest pain was described as sharp, intermittent, radiating to the left shoulder, and not associated with exertion. Additional symptoms included palpitations, orthopnea, bilateral lower extremity edema, paroxysmal nocturnal dyspnea, and dry cough. He denied lightheadedness, syncope, fever, nausea, vomiting, abdominal pain, recent infections, or other precipitating events.

The patient's medical history was notable for a presumed embolic stroke (attributed to NICMP) approximately one year ago, which resolved without residual deficit. Coronary angiography revealed normal coronary arteries at that time.

Current presentation-related medications involved apixaban, spironolactone, losartan, metoprolol tartrate, sotalol, and furosemide. However, the patient had not been compliant with medication for the past three months due to insurance-related issues.

There was no history of cigarette smoking, alcohol consumption, drug use, chemotherapy, or toxic exposures. Family history was significant for paternal hypertension, with no reported history of cardiac diseases such as genetic cardiomyopathies among siblings, parents, or grandparents.

The initial vital signs were as follows: temperature 37°C, heart rate 144 beats per minute, blood pressure 119/75 mmHg, saturation 95% on room air, and respiratory rate 22 breaths per minute. Physical examination revealed no acute distress. Cardiac auscultation identified a systolic murmur at the apex (II/VI). Jugular veins were not visible due to body habitus. Bibasilar crackles were noted on pulmonary auscultation. Peripheral pulses were symmetric, and minimal bilateral lower extremity edema was observed.

**Figure 1:**
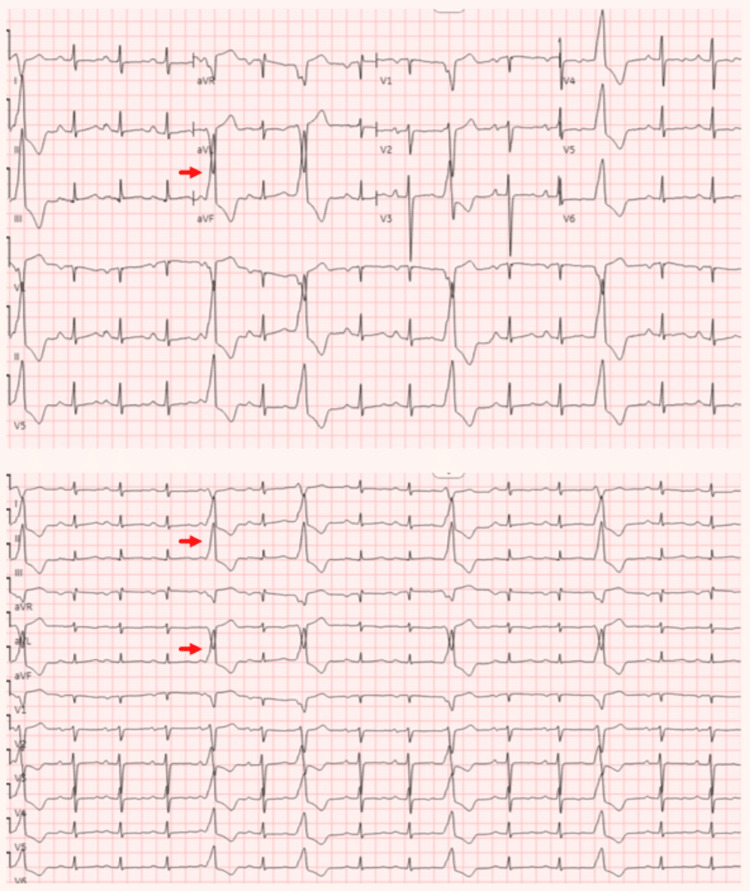
ECG and 12-lead rhythm strip The top panel is the ECG while the bottom panel is the 12-lead rhythm strip. PVCs are indicated by red arrows demonstrating LBBB pattern with positivity in the inferior leads, suggesting an origin from the RVOT. PVC: premature ventricular contractions; LBBB: left bundle branch block; RVOT: right ventricular outflow tract

The ECG revealed frequent premature ventricular contractions (PVCs) (Figure 1).

A chest X-ray showed mild pulmonary congestion. Initial laboratory results are summarized in Table 1.

**Table 1:** Initial workup results BNP: B-type natriuretic peptide; ALT: alanine transaminase; AST: aspartate aminotransferase; LDL: low-density lipoprotein; TSH: thyroid-stimulating hormone; ANA: antinuclear antibodies; N/A: not available

Laboratory parameter	Initial result	Peak result	Reference range
Troponin	29 pg/mL	N/A	<30 pg/mL
BNP	725 pg/mL	N/A	<100 pg/mL
Creatinine	1.12 mg/dL	2.3 mg/dL	0.66 - 1.25 mg/dL
ALT	37 units/L	70 units/L	0 - 49 units/L
AST	28 units/L	57 units/L	17 - 59 units/L
LDL	147 mg/dL	N/A	<100 mg/dL
Triglycerides	123 mg/dL	N/A	<150 mg/dL
Hemoglobin A1C	5.6 %	N/A	< 5.7 %
TSH	10.66 mIU/L	N/A	0.47 - 4.68 mIU/L
T4 free	2.2 ng/dL	N/A	0.6 - 2.5 ng/dL
T3 total	123 ng/dL	N/A	97 - 169 ng/dL
ANA	None detected	N/A	None detected

Transthoracic echocardiography (TTE) demonstrated severe left ventricular (LV) dilation with an ejection fraction (EF) of 10% and global hypokinesis. Additionally, there was mild dilation of the right ventricle with moderate to severe systolic dysfunction. Mild mitral regurgitation, as well as mild pulmonary hypertension (PHTN) and tricuspid regurgitation, were also noted (Video 1).

**Video 1:** Transthoracic echocardiogram Apical four-chamber view demonstrating biventricular dilation and severely reduced left ventricular ejection fraction.

**Figure 2:**
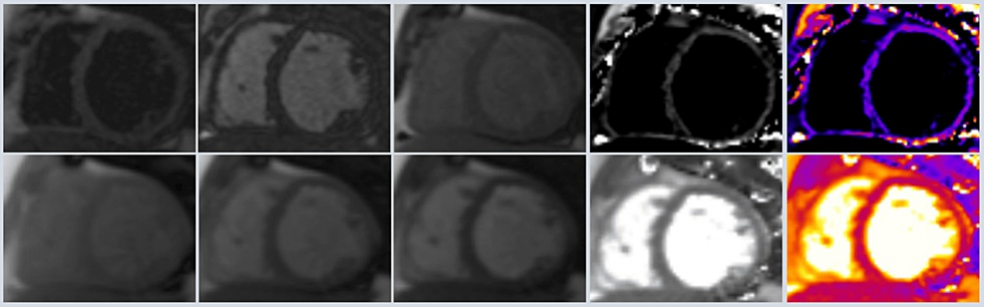
Cardiac MRI The upper and lower panels represent T1 and T2 tissue mapping, respectively, without identifiable abnormalities.

Initially, the management involved IV diuresis, apixaban, sotalol, and spironolactone. The clinical course was complicated by hypotension, acute kidney injury, and mild transaminitis (Table 1). Consequently, dobutamine was initiated, and an intra-aortic balloon pump (IABP) was inserted for the cardiogenic shock. Diuresis was continued during inotropic and mechanical cardiac support. The sotalol was subsequently switched to amiodarone. Dobutamine was gradually tapered off, and the right heart catheterization performed while the patient was on IABP revealed severe PHTN (mean pulmonary artery pressure of 44 mmHg), pulmonary capillary wedge pressure of 26 mmHg, and cardiac index (Fick method) of 1.5 L/min/m². Digoxin therapy was initiated, and subsequently, the IABP was removed. Following this, cardiac MRI (CMRI) showed no focal abnormal late gadolinium enhancement indicative of fibrous changes, scarring, or infiltrative cardiac disease. T1 and T2 parametric tissue mapping values were within normal limits, with no evidence of Fabry disease (Figure 2).

Additional important workup results are presented in Table 2.

**Table 2:** Iron studies and infectious workup HIV: human immunodeficiency virus; Ag: antigen; Ab: antibody

Laboratory parameter	Result	Reference range
Ferritin	50.3 ng/mL	17.9 - 464.0 ng/mL
Iron	63 mcg/dL	49 - 181 mcg/dL
Iron saturation	13%	20 - 50 %
HIV Ag/Ab	Nonreactive	Nonreactive
Trypanosoma cruzi antibody	Negative	Negative

The plasma amino acids, acylcarnitine, free and total carnitine, creatinine kinase, and urine organic acids were within normal ranges. The genetic panel for NICMP revealed a heterozygous pathogenic variant, c.64673-2A>G (splice acceptor), in the TTN gene associated with autosomal dominant DCM. This variant was located in the A-band region of the gene, where most DCM-related variants are typically found. The mutation identified was a splice variant expected to result in a truncated protein. Additionally, the patient was found to be heterozygous for both AKAP9 and MYH6 genes, each of uncertain significance.

Following improvement in kidney function, goal-directed medical therapy was gradually initiated and the patient was discharged on losartan, digoxin, spironolactone, aspirin, furosemide, and amiodarone.

**Figure 3:**
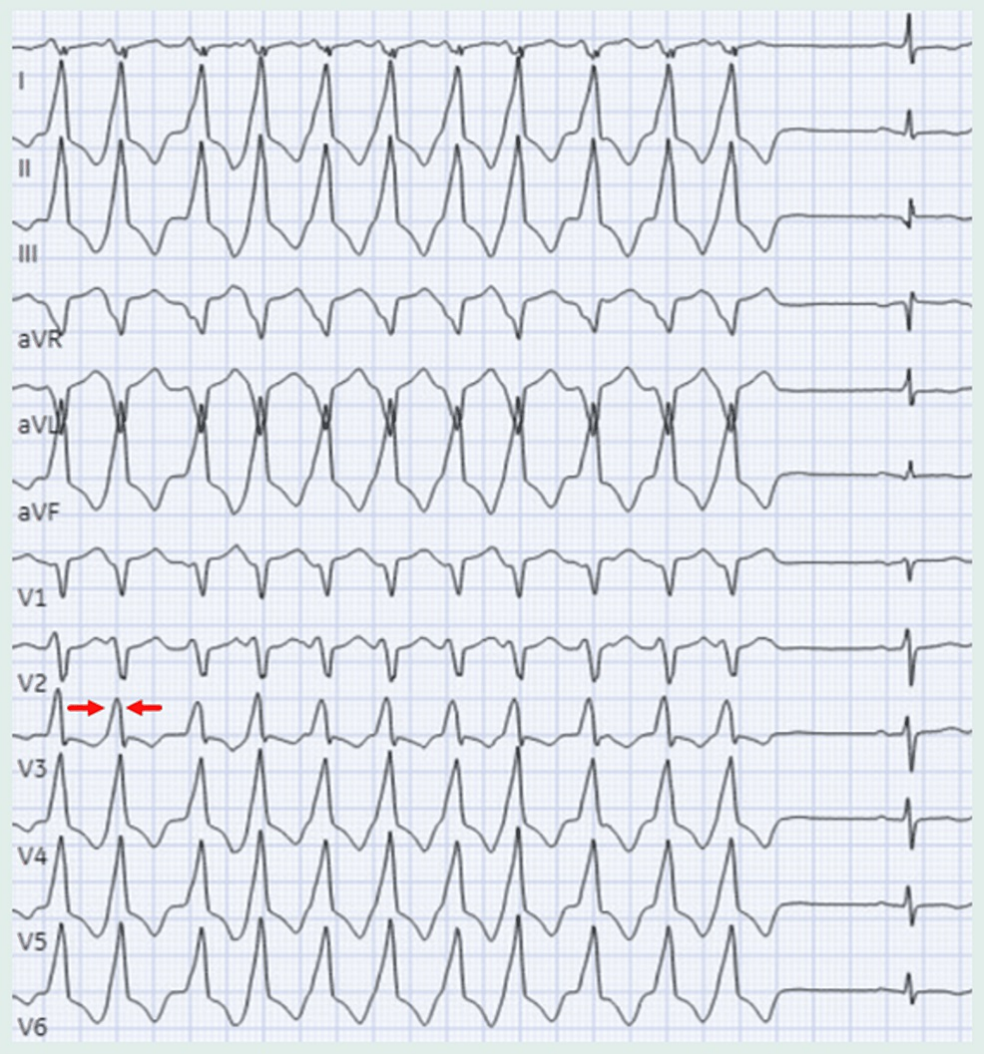
Rhythm strip during MMVT The MMVT has the same morphology as the PVCs with a transition point at V3 as indicated by the red arrows. MMVT: monomorphic ventricular tachycardia; PVC: premature ventricular contraction

After hospital discharge, an implantable cardioverter-defibrillator (ICD) was placed for primary prevention. However, given a high burden of PVCs and several runs of monomorphic ventricular tachycardia (Figure 3) requiring anti-tachycardia pacing and one shock, he was scheduled for PVC ablation in hopes of terminating runs of outflow-tract VT.

In the interim, antiarrhythmic medications were changed to dofetilide and mexiletine but failed to prevent VT episodes. Ablation attempts were then pursued, but unfortunately, were unsuccessful on two separate occasions as programmed electrical stimulation from both the RVOT and left ventricular outflow tract (LVOT) did not induce VT. The patient was discharged on propranolol, dofetilide, mexiletine, losartan, and spironolactone.

## Discussion

This presentation, characterized by compromised biventricular systolic function and dilation along with positive genetic testing is consistent with the diagnosis of TTNtv-related DCM, supported by a negative ischemic workup, the absence of valvular abnormalities and infiltrative diseases on CMRI, and a negative history of toxic and infectious exposures. The left bundle branch block (LBBB) pattern and inferior lead positivity indicate the RVOT origin of frequent PVCs [8]. In 70-80% of cases, monomorphic ventricular tachycardia (MMVT) arises from the RVOT and 15-25% from the LVOT [9,10]. The typical electrocardiographic pattern of outflow tract ventricular arrhythmias is the LBBB and inferior axis positivity. An early precordial transition (≤ V2) suggests LVOT origin, while a late transition (≥ V4) indicates RVOT origin. Ventricular arrhythmias with an intermediate transition (V3 transition) may originate from either outflow tract [11]. The rhythm strip shown in Figure 2 illustrates the electrical axis transition point in lead V3, supporting the potential origins of MMVT from both the RVOT and LVOT.

That the VT was non-inducible on two separate occasions indicates the possibility that its origin could be from epicardial, deep intramural circuits in the LV ostium, or another location.

The incidence and prevalence of idiopathic DCM were initially estimated based on a study conducted from 1975 to 1984 in Olmstead County, Minnesota, United States. According to this study, the age-adjusted and sex-adjusted incidence was six per 100,000 person-years, with a prevalence of 36.5 per 100,000 people, approximately one in 2,700 individuals [12]. Unfortunately, no other population-based studies have provided data on the frequency of idiopathic DCM. However, using surrogate approaches for assessment, the prevalence of one in 2,500 individuals is considered a significant underestimate [13].

A familial basis has been proposed for up to 50% of DCM cases [14]. Truncating variants in the gene encoding the sarcomeric protein titin have been identified as the largest single genetic contributor to DCM, accounting for approximately 25% of familial cases and 18% of sporadic cases of idiopathic DCM [3].

Titin is the largest protein in humans, expressed in cardiac and skeletal muscle, where it plays a crucial role in sarcomere structure and function. It is pivotal in maintaining sarcomere integrity, force transmission, stretch sensing, and signaling [15]. Truncating mutations in the TTN gene result in shortened titin, leading to the development of DCM.

Patients with TTNtv-related DCM have nearly three times higher odds of developing early-onset atrial and VT compared to those without TTNtv [7]. In one cohort of DCM patients followed long-term, those with TTNtv experienced increased ventricular arrhythmias compared to other forms of DCM, although survival rates were similar. In these patients, arrhythmias manifested predominantly in association with additional triggering factors such as viral infections, cardiac inflammation, systemic diseases, or toxic exposures [16].

Another contributing factor to malignant arrhythmic events in patients with TTNtv-related DCM is reduced left ventricular ejection fraction (LVEF). The majority of these events occur in patients with severely reduced LVEF, supporting the consensus for ICD implantation in patients with LVEF <35% for primary prevention [17]. Importantly, 10% of patients with preserved LVEF develop atrial fibrillation or non-sustained VT, underscoring the necessity for regular Holter monitoring in all patients with TTNtv [17].

Myocardial fibrosis serves as a substrate for ventricular arrhythmias in patients with DCM, and mid-wall fibrosis is specifically linked to cardiac death and appropriate ICD therapy [18]. The prevalence of mid-wall fibrosis in patients with TTNtv-related DCM is not statistically different compared to those without TTNtv [19,20]. However, the combination of TTNtv and mid-wall fibrosis significantly increases the risk of ventricular arrhythmias, whereas their absence is associated with lower risk [19]. Thus, the presence of TTNtv and mid-wall fibrosis may aid in risk stratification for decisions regarding ICD placement.

Another potential factor contributing to the increased susceptibility to ventricular arrhythmias in TTNtv-related DCM is increased LV wall stress due to thinner LV walls and lower indexed LV mass for a similar degree of dilation [19,20]. Increased wall stress is associated with a greater risk of ventricular arrhythmias through the promotion of triggered activity via early and delayed afterdepolarizations and through the facilitation of reentry circuits via shortening the effective refractory period with increased dispersion [21,22].

Danicamtiv is an emerging targeted therapy for TTNtv-related DCM enhancing myocardial contractility through increased myosin recruitment [23]. While optimal guideline-directed medical therapy typically leads to LV reverse remodeling in the majority of patients, some experience late deterioration in LVEF [17]. Managing VTs presents challenges, as 53% of patients experience VT recurrence following ablation [24].

## Conclusions

In summary, TTNtv mutation should always be considered in the differential diagnosis of patients with DCM. Genetic testing is imperative for DCM workup. TTNtv-related DCM represents an independent risk factor for VT originating from the outflow tracts. While VT most commonly originates from the RVOT and less frequently from the LVOT, in rare instances, the delineation of the substrate is unsuccessful, suggesting potential epicardial, deep intramural, or other uncommon sources of VT.
